# The Role of Immune Cells and Signaling Pathways in Diabetic Eye Disease: A Comprehensive Review

**DOI:** 10.3390/biomedicines12102346

**Published:** 2024-10-15

**Authors:** Vincenzo Barone, Pier Luigi Surico, Francesco Cutrupi, Tommaso Mori, Gabriele Gallo Afflitto, Antonio Di Zazzo, Marco Coassin

**Affiliations:** 1 Department of Ophthalmology, Campus Bio-Medico University, 00128 Rome, Italy; vincenzo.barone@unicampus.it (V.B.); francesco.cutrupi@unicampus.it (F.C.); tmori@health.ucsd.edu (T.M.); a.dizazzo@policlinicocampus.it (A.D.Z.); m.coassin@policlinicocampus.it (M.C.); 2 Ophthalmology Operative Complex Unit, Campus Bio-Medico University Hospital Foundation, 00128 Rome, Italy; 3 Schepens Eye Research Institute of Massachusetts Eye and Ear, Harvard Medical School, Boston, MA 02114, USA; 4 Department of Ophthalmology, University of California San Diego, La Jolla, CA 92122, USA; 5 Ophthalmology Unit, Department of Experimental Medicine, University of Rome “Tor Vergata”, 00128 Rome, Italy; gabrielegalloafflitto@gmail.com; 6 Moorfields Eye Hospital NHS Foundation Trust, London EC1V 2PD, UK

**Keywords:** diabetes, diabetic eye disease, pathophysiology, diabetic retinopathy, diabetic keratopathy, immune cells, inflammation, signaling pathways

## Abstract

Diabetic eye disease (DED) encompasses a range of ocular complications arising from diabetes mellitus, including diabetic retinopathy, diabetic macular edema, diabetic keratopathy, diabetic cataract, and glaucoma. These conditions are leading causes of visual impairments and blindness, especially among working-age adults. Despite advancements in our understanding of DED, its underlying pathophysiological mechanisms remain incompletely understood. Chronic hyperglycemia, oxidative stress, inflammation, and neurodegeneration play central roles in the development and progression of DED, with immune-mediated processes increasingly recognized as key contributors. This review provides a comprehensive examination of the complex interactions between immune cells, inflammatory mediators, and signaling pathways implicated in the pathogenesis of DED. By delving in current research, this review aims to identify potential therapeutic targets, suggesting directions of research for future studies to address the immunopathological aspects of DED.

## 1. Introduction

Diabetes mellitus (DM) encompasses a range of metabolic diseases characterized by persistent high blood sugar levels, resulting from either the inadequate production of insulin, ineffective insulin action, or both [1]. Currently, DM represents a critical global health issue, affecting over 537 million people worldwide, or 10.5% of individuals between 20 and 79 years of age. This number is anticipated to rise to 783 million (12.2%) by 2045 [2].

Chronic hyperglycemia in diabetic individuals is associated with long-term damage and dysfunction across various organs, including the eyes, nerves, kidneys, cardiovascular system, and blood vessels. DM is broadly divided into two primary types based on its etiopathogenesis. Type 1 diabetes, accounting for approximately 5–10% of cases, arises from the autoimmune destruction of pancreatic β-cells, resulting in a total lack of insulin production. In contrast, type 2 diabetes, which represents 90–95% of cases, is primarily caused by insulin resistance and often accompanied by a relative deficiency in insulin secretion [1].

**Figure 1:**
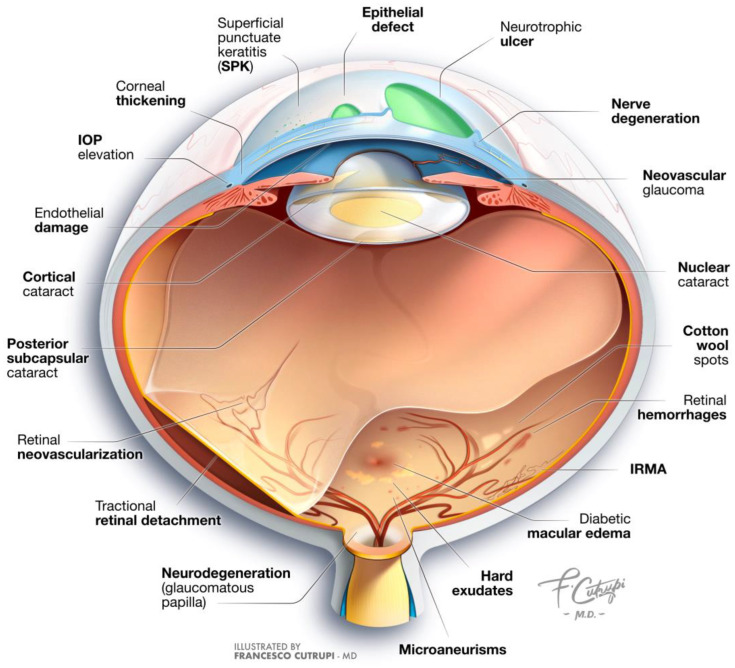
**Clinical manifestations of diabetic eye disease.** This illustration provides a comprehensive overview of the ocular signs associated with diabetes mellitus. It highlights key manifestations including corneal damage, cataract formation, retinal degeneration, and the development of glaucoma (IOP: intraocular pressure; IRMAs: intraretinal microvascular abnormalities).

Diabetic eye disease (DED) is the most common complication of DM. When using the term DED in this review, we aim to encompass a variety of ocular conditions, including diabetic retinopathy (DR), diabetic macular edema (DME), diabetic keratopathy (DK), diabetic cataract, and glaucoma (Figure 1) [3].

DED is a major contributor to visual impairment and blindness, especially among working-age adults in high-income countries [4]. The risk of developing DED is influenced by several factors, such as poor glycemic control, the presence of cardiovascular comorbidities, disease duration, and the type of diabetes, with type 1 diabetes carrying a higher risk [5]. Despite significant scientific progress in recent decades, the pathophysiological mechanisms underlying DED remain only partially understood. Recent studies have shown that patients with diabetes, particularly those with DED, exhibit a range of inflammatory cytokines, chemokines, altered signaling pathways, and immune cell dysfunctions [3,6,7,8,9].

This review aims to provide a comprehensive examination of the complex interactions between the immune cells, inflammatory mediators, and signaling pathways involved in the pathogenesis of DED. Emerging evidence highlights the role of immune-mediated processes in the development and progression of DED, contributing to the chronic inflammation that characterizes these conditions. By synthesizing current research, this review seeks to elucidate potential therapeutic targets and outline directions for future studies aimed at addressing the immunological aspects of DED.

## 2. Methods

The review was conducted using PubMed (https://pubmed.ncbi.nlm.nih.gov) and Reference Citation Analysis (RCA) (https://www.referencecitationanalysis.com). PubMed, a well-established and widely trusted biomedical literature database maintained by the National Library of Medicine (NLM), was chosen as the primary resource for this research due to its extensive coverage of peer-reviewed journals on medicine and life sciences. The search strategy involved using a combination of search terms, including “Immune Cells,” “Signaling Pathways,” “Inflammation,” and terms related to diabetic eye diseases such as diabetic retinopathy (DR), diabetic macular edema (DME), cataract, keratopathy, corneal nerves, and glaucoma. Additional terms like “dendritic cells,” “macrophages,” “T cells,” and “cytokine signaling” were incorporated to cover a broader range of the immune-related components involved in diabetic eye disease. Boolean operators (AND, OR, NOT) were employed to structure the search and ensure the comprehensive retrieval of the relevant literature while minimizing irrelevant results.

The search was limited to articles written in English to ensure clarity and accessibility. The titles and abstracts of the retrieved articles were manually screened to identify those relevant to the study objectives. Full-text articles were reviewed to extract detailed information on the involvement of immune cells, molecular signaling pathways, and their contribution to the pathophysiology of diabetic eye conditions, including macular edema, cataract, keratopathy, corneal nerve degeneration, and glaucoma. Furthermore, the molecular mechanisms of diabetic retinopathy and the role of immune signaling pathways in retinal neurodegeneration were closely examined.

To supplement electronic searches, manual reviews of reference lists and citation tracking from key articles were conducted to ensure thorough coverage. This search strategy aimed to provide a comprehensive understanding of the role of immune cells and signaling pathways in diabetic eye disease, particularly focusing on immune responses, molecular cascades, and their implications for the development and progression of diabetic retinopathy, macular edema, cataract, keratopathy, and glaucoma.

## 3. Pathophysiology of Diabetic Retinopathy

DR is a microvascular complication of DM, primarily driven by prolonged hyperglycemia, which induces both structural and functional changes in the retinal vasculature [10,11]. DR is one of the most common ocular complications of diabetes, affecting around 20–40% of individuals with DM. However, the lifetime risk of DR is significantly higher, reaching approximately 60% in patients with type 2 DM and up to 90% in those with type 1 DM [12,13,14]. Therefore, an early diagnosis is essential for preventing progression and complications [15].

Several factors are consistently linked to the development of DR, including long-standing diabetes, inadequate glycemic control, and hypertension [12,13]. Additional systemic factors, such as dyslipidemia, diabetic nephropathy, obesity, anemia, and markers of systemic inflammation and endothelial dysfunction, also contribute to the development of DR [16,17]. Ocular factors, such as a history of cataract surgery, have been associated with the progression of DR and the development of DME [18]. Conversely, myopia appears to offer some protective effects against DR [19].

**Figure 2:**
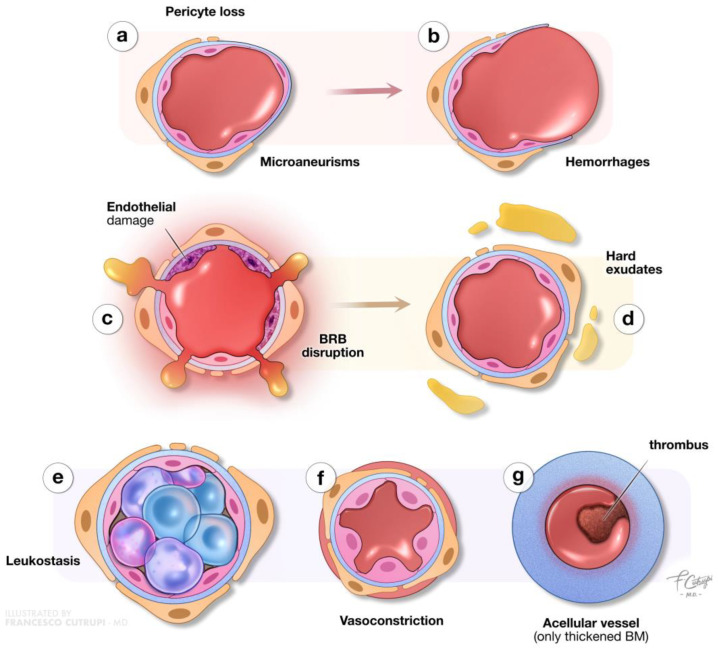
**Microvascular alterations in non-proliferative diabetic retinopathy.** This illustration details the microvascular changes occurring in non-proliferative diabetic retinopathy. Chronic hyperglycemia results in the loss of pericytes in retinal capillaries, undermining structural support and leading to the formation of microaneurysms (**a**) and vessel ruptures accompanied by hemorrhages (**b**). Strong inflammatory processes, along with endothelial and basement membrane (BM) alterations, compromise the blood–retinal barrier (BRB), increasing vascular permeability (**c**) and resulting in edema and hard exudates (**d**). Furthermore, the retinal blood flow is strongly compromised due to leukostasis (with vascular occlusion), vasoconstriction, and the presence of highly thrombogenic acellular capillaries. Furthermore, the retinal-blood-flow is-strongly-compromised due to leukostasis (with-vascular occlusion) (**e**), vasoconstriction (**f**), and the presence of-highly-thrombo-genic acellular capillaries (**g**).

Clinically, DR is categorized into two primary stages: non-proliferative diabetic retinopathy (NPDR) and proliferative diabetic retinopathy (PDR). NPDR, the initial stage, is characterized by specific microvascular abnormalities such as microaneurysms, intraretinal hemorrhages, and hard exudates (Figure 2) [10].

The central role of hyperglycemia in NPDR lies in its activation of various pathways, including the polyol pathway, the accumulation of advanced glycation end products (AGEs), the protein kinase C pathway, the hexosamine pathway, the vascular endothelial growth factor (VEGF) pathway, the nuclear factor kappa-B (NF-κB) pathway, and the Janus Kinase—Signal Transducer and Activator of Transcription JAK-STAT pathway. [11,20,21,22,23] A critical early event in NPDR is the loss of pericytes, which are vital for maintaining the structural integrity of retinal capillaries [24]. The hyperglycemia-induced apoptosis of pericytes weakens capillary walls, leading to the formation of microaneurysms and intraretinal hemorrhages, which are among the earliest detectable clinical signs of NPDR [25]. Furthermore, basement membrane thickening and endothelial cell damage lead to the breakdown of the blood–retinal barrier (BRB), increasing vascular permeability [26].

In the early stages of microvascular impairment, key inflammatory cytokines, including VEGF, Interleukin-1β (IL-1β), Tumor Necrosis Factor- α (TNF-α), and Interleukin-6 (IL-6), are primarily secreted by Müller cells, microglia, and the retinal pigment epithelium (RPE) [27,28]. IL-6 plays a role in disrupting astrocyte function, which compromises the inner blood–retinal barrier (BRB), while Interleukin-8 (IL-8) and Monocyte Chemoattractant Protein-1 (MCP-1) promote the infiltration of immune cells such as neutrophils and monocytes into the retina [29,30]. This chronic inflammation leads to leukocyte adhesion to the vascular endothelium, a process known as leukostasis, which further weakens the BRB and exacerbates vascular permeability and retinal non-perfusion, resulting in the clinical presence of hard exudates [31,32,33,34]. IL-1β activates the NF-κB pathway, leading to the increased production of IL-6 and IL-8. It also activates caspase-1, which promotes apoptosis [28]. TNF-α promotes pericyte loss and capillary degeneration, further accelerating the progression of DR [35,36]. Other inflammatory molecules, including inducible Nitric Oxide Synthase (iNOS) and Cyclooxygenase-2 (COX-2), amplify the inflammatory cascade, while Matrix Metalloproteinase (MMPs), particularly MMP-2 and MMP-9, regulate inflammation and tissue remodeling, playing significant roles in retinal neovascularization [37,38].

As NPDR progresses, endothelial damage intensifies, creating an imbalance between vasodilatory and vasoconstrictive factors. This imbalance favors vasoconstriction through molecules such as endothelin and thromboxane A2, which exacerbate retinal ischemia by creating a hypoxic environment. In advanced NPDR, widespread endothelial cell loss results in capillary occlusion, with capillaries reduced to thrombogenic, thickened basement membrane tubes. Clinically, this stage is marked by the appearance of cotton wool spots as a result of retinal ischemia and intraretinal microvascular abnormalities (IRMAs) [10,31].

Recent research has identified retinal neurodegeneration as another critical factor in DR progression [39]. The apoptosis of retinal neurons, documented in diabetic patients, precedes the clinical signs of DR and can be detected using spectral-domain optical coherence tomography (SD-OCT) [40,41]. This process is further exacerbated by mitochondrial dysfunction, oxidative stress, and endoplasmic reticulum (ER) stress [42,43]. Additionally, extracellular glutamate accumulation and a decrease in neuroprotective factors, such as pigment epithelium-derived factor (PEDF), somatostatin, and neurotrophins, contribute to the neurodegeneration in DR [39].

PDR, the advanced stage of DR, is characterized by the formation of new abnormal blood vessels, a phenomenon known as neovascularization. This process is triggered by chronic retinal ischemia, primarily driven by hypoxia-inducible factors (HIFs). Under hypoxic conditions, HIFs stimulate the overproduction of VEGF, causing an imbalance between pro-angiogenic and anti-angiogenic factors [10,44,45]. This imbalance leads to the growth of fragile and dysfunctional blood vessels on the surface of the retina and optic disc, which are structurally weak and susceptible to rupture and bleeding [44,45]. In addition to VEGF, other angiogenic mediators such as phospholipase A2 (PLA2), insulin-like growth factor I (IGF-1), hepatocyte growth factor (HGF), basic fibroblast growth factor (b-FGF), platelet-derived growth factor (PDGF), and angiopoietins further contribute to the vascular instability seen in PDR [44,46].

These vessels often extend into the vitreous body, where they become anchored by fibrovascular tissue [47]. Over time, this fibrovascular tissue can contract, leading to tractional retinal detachment, a severe complication that significantly impairs vision [44]. Tractional retinal detachment and vitreous hemorrhage are hallmark features of advanced PDR, representing sight-threatening conditions that, if untreated, can result in severe visual loss [10].

## 4. Pathophysiology of Diabetic Macular Edema

DME is the primary cause of vision impairment in diabetic patients, and its global prevalence is increasing [48]. DME can occur at any stage of DR, whether in its non-proliferative or proliferative phases. When macular thickening affects or threatens the fovea, patients may experience significant visual disturbances, including metamorphopsia and vision loss [49,50]. The prevalence of DME varies widely, ranging from 4% to 14% in individuals with type 1 diabetes and from 1% to 5% in those with type 2 diabetes [51,52]. Numerous risk factors are associated with DME and DR, such as a prolonged diabetes duration, poor glycemic control, hypertension, hyperlipidemia, and genetic predisposition [53].

The pathogenesis of DME shares many mechanisms with DR, and particularly the vascular damage driven by hyperglycemia, inflammation, oxidative stress, and neurodegeneration [30,48]. Together, these factors disrupt fluid regulation in the retina, leading to an imbalance between fluid influx and drainage by Müller cells and the RPE. This ultimately results in the accumulation of intraretinal fluid or subretinal fluid [48,54,55,56].

Fluid regulation in a healthy retina is dependent on the integrity of the BRB and the active drainage function of Müller cells and the RPE [48,56,57,58]. These glial cells are crucial in maintaining the retinal extracellular environment, and particularly in regulating fluid and ion balance [48,59,60,61]. Equipped with aquaporins, ion channels, and other proteins, Müller cells facilitate the removal of excess fluid from the retina into the vitreous or vascular system. However, in the diabetic retina, chronic hyperglycemia and metabolic imbalances impair the ability of Müller cells to drain fluid effectively, resulting in fluid retention and cytotoxic swelling [50,62,63,64,65]. This dysfunction is further complicated by the structural changes in Müller cells under diabetic conditions, such as the swelling of their apical processes [48,66,67,68]. This form of edema exacerbates neurotoxicity, contributing to worsening vision loss and increased extracellular fluid volume [48,56].

Extracellular, or vasogenic, edema arises from the breakdown of the BRB, leading to increased vascular permeability and fluid leakage into extracellular retinal spaces [61]. In NPDR, BRB disruption and subsequent DME are primarily driven by the activation of VEGF pathways and inflammatory processes, which upregulate cytokines and growth factors that promote vascular permeability [69,70].

## 5. Pathophysiology of Diabetic Keratopathy

**Figure 3:**
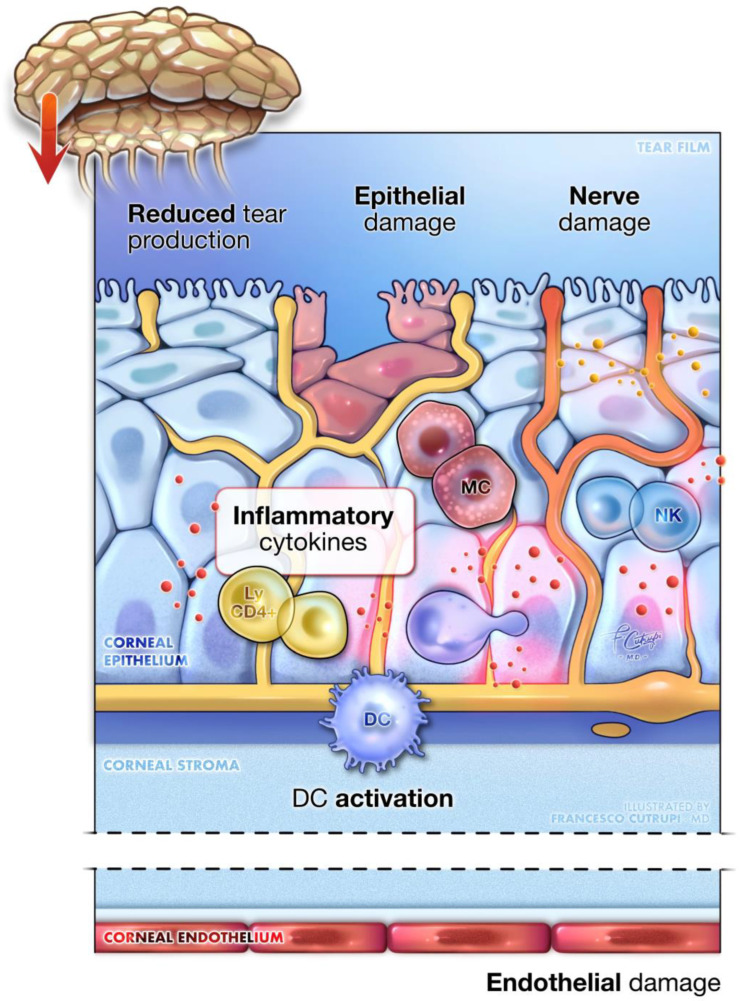
Diabetic keratopathy. A detailed close-up of corneal tissue showcases the main pathogenic mechanisms underlying diabetic keratopathy. The image illustrates the epithelial dysfunction seen, alongside pronounced inflammation, mediated by immune cells within the cornea. Additionally, it depicts damage to the corneal nerves and lacrimal gland innervation, leading to decreased sensitivity, neurotrophic alterations, and reduced tear production. Endothelial cell damage is also present, leading to corneal edema and thickening. Key inflammatory cells include the activation of dendritic cells (DC), mast cells (MC), natural killer lymphocytes (NK), and resident memory CD4⁺ lymphocytes (ly CD4⁺).

DK is a degenerative corneal condition that may impact as many as 70% of individuals with diabetes [71]. This disorder is characterized by several corneal abnormalities, including increased corneal thickness, epithelial damage, delayed wound healing, endothelial dysfunction, and reduced corneal sensitivity [71,72,73]. Clinically, DK presents with various manifestations such as superficial punctate keratitis, recurrent corneal erosions, persistent epithelial defects, and ulcers, which can cause significant visual impairment in their advanced stages [74,75,76,77] (Figure 3).

The pathophysiology of DK is complex and multifactorial, with hyperglycemia-induced oxidative stress and inflammation playing central roles in driving cellular damage and neurodegeneration. The corneal epithelium is particularly affected due to its insulin-independent glucose uptake, which, under diabetic conditions, leads to excessive glucose influx and AGE accumulation [78,79,80]. This process promotes oxidative stress and impairs cellular function [80,81].

The increased oxidative stress activates NF-κB, initiating inflammatory responses that further delay epithelial regeneration and wound healing [82]. Additionally, AGEs stimulate Nicotinamide Adenine Dinucleotide Phosphate (NADPH) oxidase activity, increasing the production of reactive oxygen species (ROS) and promoting apoptosis, particularly in corneal endothelial cells [83]. Hyperglycemia also elevates diacylglycerol (DAG) levels, leading to the activation of protein kinase C, which amplifies ROS production and disrupts protective cellular pathways such as the EGFR-PI3K/Akt pathway, which is crucial for cell survival and wound repair [84,85,86]. Additionally, transforming growth factor-Beta (TGF-β), activated by oxidative stress and AGEs, contributes to fibrogenesis and hinders re-epithelialization, further complicating the healing process [87,88]. Another significant pathological feature of DK is pyroptosis, a form of programmed cell death triggered by metabolic stress through the activation of the NLRP3 inflammasome in corneal cells [89]. This process activates caspase-1, releasing pro-inflammatory cytokines such as IL-1β and IL-18, which exacerbate inflammation and corneal damage [90,91,92].

In addition to these processes, diabetic corneas show an increased presence of resting mast cells, activated natural killer (NK) cells, and memory CD4+ T cells, which respond to cellular stress and damage, further contributing to the inflammatory environment [93,94]. The corneal stroma, endothelium, and tight junctions are also affected by DK. Corneal endothelial cells, in particular, are highly susceptible to oxidative damage, leading to decreased cell density and impaired barrier functions [95,96,97,98]. This vulnerability is compounded by a weakened antioxidant response, mediated by the Nrf2 signaling pathway, which is crucial for protecting cells against oxidative stress [96,97,98].

Hyperglycemia also disrupts the structural integrity of the cornea. The loss of essential structural proteins like laminin-5 weakens its barrier function and slows wound healing [86,99,100,101]. Furthermore, it damages the nerve fibers that innervate the lacrimal gland, reducing tear secretion, destabilizing the tear film, and promoting dry eye disease. This dysfunction impairs corneal health, increases the risk of infections, and further compromises ocular surface integrity in diabetic patients [102,103,104]. This neuropathy also disrupts sensory and trophic functions, making the eye more prone to injury and delaying the healing process, potentially leading to neurotrophic ulcers and, eventually, corneal perforation [105,106]. Neurotrophic factors, such as nerve growth factor (NGF) and neurotrophin-3 (NT-3), which are vital for corneal health, become dysregulated under diabetic conditions, worsening the progression of keratopathy [107].

## 6. Pathophysiology of Diabetic Cataract

Cataract is a leading cause of visual impairment, affecting both the general population and individuals with diabetes [108]. However, the incidence rate of cataracts in diabetic patients is 20.4 per 1000 person-years, significantly higher than the 10.8 per 1000 person-years observed in the general population [109,110,111].

Diabetic cataracts present in three main forms: cortical, nuclear, and posterior subcapsular (PSC), which often coexist in the same individual. Among patients with type 2 diabetes, approximately 65% develop cortical cataracts, 48% experience nuclear cataracts, and 42% develop a PSC cataract. Notably, while cortical cataracts are generally not strongly correlated with blood glucose fluctuations, PSC cataracts are closely associated with poor glycemic control [112].

The pathogenesis of diabetic cataracts remains only partially understood. Cataractogenesis is likely driven by multiple interacting pathways, with glucose metabolism playing a central role. Glucose enters the lens from the aqueous humor and is converted into sorbitol via the polyol pathway [113,114]. Elevated blood glucose levels lead to an increase in glucose concentration within both the aqueous humor and the lens. Due to limited membrane permeability, sorbitol accumulates within lens cells, causing osmotic imbalance and cellular swelling, which ultimately lead to lens opacity [115,116].

Moreover, the polyol pathway depletes NADPH, a critical cofactor required to maintain the cellular redox balance and antioxidant defenses, particularly for the regeneration of glutathione. As NADPH levels drop, the lens loses its ability to neutralize ROS, thereby exacerbating oxidative stress and cellular damage [117]. Oxidative stress plays a crucial role in cataract formation in diabetes [113]. One key pathway involves glucose auto-oxidation and mitochondrial dysfunction, both of which significantly elevate ROS production. Additionally, the non-enzymatic glycation of proteins results in the formation of AGEs, which bind to their receptors on cells, triggering intracellular signaling pathways, including the NF-κB pathway [118,119]. NF-κB, a pivotal transcription factor, regulates genes associated with inflammation and cellular stress responses, further intensifying oxidative stress, as indicated by the higher levels of pro-inflammatory cytokines in the lens epithelial cells of diabetic patients compared to non-diabetic individuals [120,121]. Additionally, impaired autophagy, a cellular process responsible for removing damaged proteins and organelles, leads to the accumulation of dysfunctional cellular components [122,123,124].

This oxidative environment and impaired cellular function damage essential biomolecules, such as lipids, proteins, and DNA, within the lens, leading to increased light scattering and directly contributing to lens opacity [118,119,125].

## 7. Pathophysiology of Diabetic Glaucoma

Open-angle glaucoma (OAG) is a multifactorial optic neuropathy characterized by the progressive degeneration of retinal ganglion cells (RGCs), which leads to visual field loss. Globally, it is the second leading cause of blindness. Numerous meta-analyses have suggested an increased risk of OAG in individuals with diabetes, though the precise nature of this relationship remains a subject of debate [126,127,128,129].

Some hypotheses propose that diabetes may contribute to microvascular damage or a reduction in the nutrient supply to retinal ganglion cells (RGC) axons, potentially due to disrupted blood flow regulation in the optic nerve head [130,131]. Several studies have shown that intraocular pressure (IOP) tends to be higher in diabetic patients compared to non-diabetic patients, potentially due to impaired autonomic function and microvascular injury; however, other evidence does not show any significant correlation between the two [132,133,134,135]. Moreover, other studies demonstrate that vascular autoregulation in the retinal, choroidal, and retrobulbar circulations may be impaired in glaucoma patients, complicating the vascular contribution to glaucoma in individuals with diabetes [136,137,138,139]. Additionally, recent findings suggest that endothelial impairment plays a critical role in the development of angiopathy in DM, potentially exacerbating vascular autoregulatory issues in diabetic patients with glaucoma [140,141].

Neovascular glaucoma (NVG) is a secondary form of glaucoma characterized by the formation of abnormal blood vessels in the iris (neovascularization of the iris, NVI) and the anterior chamber angle (neovascularization of the angle, NVA) [142]. NVG often arises as a result of ischemic ocular conditions, including PDR. Retinal ischemia and hypoxia drive the release of angiogenic factors such as VEGF, which promotes neovascularization. Although NVG is relatively rare, with a prevalence of approximately 3.9%, it can cause severe glaucomatous optic neuropathy and lead to blindness [143,144]. Unlike central retinal vein occlusion, where NVG typically develops within 100 days, the progression of hypoxia and ischemia in diabetic retinopathy is slower [142].

Normally, a balance exists between pro-angiogenic and anti-angiogenic factors, but, in diabetic eyes, retinal hypoxia stimulates the release of various angiogenic factors such as VEGF, hepatocyte growth factor (HGF), hypoxia-inducible factor 1 alpha (HIF1a), insulin-like growth factor (IGF) TNF, and inflammatory cytokines like IL-1β, IL-6, and IL-8. [145,146,147,148] This shift, if severe, leads to the formation of neovascular membranes in the retina, iris, and anterior chamber angle, which obstruct the trabecular meshwork and increase IOP, eventually causing visual impairment [145].

VEGF, which is synthesized by retinal Müller cells, RPE, pericytes, ganglion cells, and non-pigmented ciliary epithelial cells, is a key contributor to the development of NVG [149]. Increased VEGF levels have been identified in the aqueous humor of diabetic patients with NVG, particularly following ocular surgeries, potentially aiding its diffusion into the anterior chamber [142]. Elevated VEGF levels disrupt the BRB by enhancing leukocyte adhesion to endothelial cells, which intensifies inflammation and causes further tissue damage [150,151]. In addition to VEGF, other factors, such as TGF-β and fibroblast growth factors (FGFs), contribute to fibroblast proliferation and the formation of fibrovascular membranes in the anterior chamber [152,153].

Chronic inflammation plays a significant role in the pathogenesis of NVG secondary to DR, as indicated by the elevated levels of inflammatory cytokines such as TNF-α, IL-6, IL-8, and IL-1β in the vitreous of diabetic patients with DR [28,154]. In this inflammatory microenvironment, Müller cells, microglia, astrocytes, and T cells become activated, further releasing pro-inflammatory factors such as TNF-α, IL-6, IFN-γ, MCP-1, and VEGF. These factors contribute to endothelial damage and the disruption of the BRB, further promoting neovascularization [28,155,156].

## 8. Immune Cells in DED

The ocular system is known for its immune privilege, which preserves visual function by managing a robust immunological defense and stringent immune surveillance to minimize local inflammatory responses [157,158,159]. The retina is embryologically linked to the brain and can be considered the brain’s window. Retinal tissue benefits from the eye’s immune privilege, which allows retinal neurons to be protected from immunogenic insults, given their poor capacity for self-renewal and survival [158]. This immune privilege is maintained by various mechanisms. The physical protections represented by the BRB and blood–aqueous barrier contribute to the isolation of the ocular system from exposure to systemic insults [160]. Endothelial, immune, and retinal cells play an active role in maintaining the ocular immune sanctuary by inhibiting effector T-cells and inflammation through the release of numerous immunomodulatory factors [158]. Antigens entering the eye spaces (i.e., vitreous cavity, subretinal space, anterior chamber) cause anterior chamber-associated immune deviation (ACAID), which serves as an active mechanism of surveillance to preserve the eye’s immune privilege [161,162].

The ocular surface also exhibits immune privilege, facilitated by the cornea’s structural features such as its avascularity, soluble immunomodulatory molecules like programmed death ligand-1 (PD-L1), resident immune cells, and mechanisms that promote a tolerance to ocular antigens, which are all complemented by neuroimmune interactions [157,159,163].

The disruption of immune privilege mechanisms in the retina and ocular surface plays a key role in the development of ocular diseases like DED. While barriers and immunosuppressive factors protect retinal neurons, dysregulated immune responses contribute to chronic inflammation and damage to both the retina and ocular surface.

In the retina, microglia derived from yolk sac primitive macrophages are key to maintaining ocular homeostasis and regulating immune privilege. These cells constantly monitor their surroundings, extending and retracting processes and performing essential tasks such as the phagocytosis of retinal debris, synaptic modulation, and supporting neighboring cells [164,165,166,167]. Initially, ameboid microglia release anti-inflammatory cytokines such as IL-4, IL-10, and IL-13, which help resolve inflammation and support neuronal survival [168]. However, in DM, microglial activation occurs when diabetic products bind to damage-associated molecular pattern (DAMP) and pathogen-associated molecular pattern (PAMP) receptors. This activation triggers microglial proliferation and morphological changes, which enhance the immune response and contribute to the progression of DR [169,170]. Activated microglia release various inflammatory mediators, including IL-1β, TNF-α, IL-6, IFN-γ, MCP-1, and VEGF. These factors induce endothelial damage, impair the BRB, and lead to neurodegeneration, further recruiting immune cells such as astrocytes and other glial cells, thereby perpetuating inflammation [171,172,173,174]. Additionally, activated microglia phagocytose apoptotic neurons, contributing to both structural and functional abnormalities in the diabetic retina [175]. In DME, microglial activation is observed especially in the subretinal space, where the microglia penetrate the basement membranes of capillaries, phagocytosing endothelial cells and facilitating BRB breakdown, which promotes DME formation [50,176,177].

Other innate immune cells, including perivascular macrophages, hyalocytes, and dendritic cells, may also contribute to retinal immune regulation. Perivascular macrophages, located between the inner BRB and the glial limitans, act as a pseudo-barrier for foreign proteins and secrete chemotactic and fibrotic factors such as leukotrienes and fibronectin [178,179,180,181]. Hyalocytes, macrophages derived from bone marrow and found at the vitreoretinal interface, are believed to play roles in antigen presentation and immunomodulation, impacting both local and systemic immune responses in diabetic eyes [182,183]. Macrophages influence cellular proliferation by producing growth factors like VEGF, PDGF, FGF, and TGF-β [184]. However, in DR, macrophages exhibit impaired phagocytic function while excessively secreting inflammatory cytokines (IL-1β, TNF-α, IL-6, and IL-12) via the NF-κB pathway, worsening inflammation [185,186].

While the focus in DR has historically been on innate immunity, increasing evidence points to the critical role of adaptive immunity in metabolic inflammation and DR progression [187]. Clinical studies have identified elevated levels of CD4+ T follicular helper (Tfh) cells in the circulation of DR patients, and mouse models have shown a link between Tfh cell upregulation and DR pathogenesis [188,189]. Inhibiting Bcl-6, a transcription factor essential for Tfh cell development, has been shown to reduce Tfh cell activity and their IL-21 cytokines, improving vascular outcomes in DR models [190]. Furthermore, increased densities of CD4+ T cells, CD8+ T cells, and CD19+ B cells have been found in the fibrovascular membranes of patients with PDR, highlighting the role of lymphocytes in DR progression [191,192]. Adaptive immunity also influences microglial activity in the retina under diabetic conditions. For example, IFN-γ from activated Th1 cells promotes pro-inflammatory responses in the microglia, while Th2 cells produce anti-inflammatory cytokines that promote alternative macrophage activation [193,194].

Also, diabetes disrupts the ocular system’s immune privilege at the cornea by promoting an immunostimulatory phenotype in corneal myeloid cells, which are located near the sub-basal nerves [195]. These cells, under diabetic conditions, become activated and express elevated levels of pro-inflammatory cytokines such as TNF-α and IL-1β. The close proximity of corneal myeloid cells to the sub-basal nerves allows for significant neuronal–immune crosstalk, which is critical for maintaining normal corneal function [196,197]. In diabetes, this interaction leads to immune-mediated damage to the corneal nerves, resulting in reduced sensitivity, a common feature of DK [195,198].

Additionally, in NVG, alongside microglial involvement in BRB impairment and VEGF release, studies have shown significantly elevated levels of white blood cells, neutrophils, and monocytes in NVG patients compared to controls, further emphasizing the importance of immune dysregulation in DED [199].

## 9. Overview of Key Signaling Pathways

Numerous signaling pathways are implicated in the development and progression of DED. One key pathway is the VEGF pathway, which is instrumental in angiogenesis. VEGF is primarily secreted by various retinal cells, including pigmented epithelial cells, pericytes, astrocytes, Müller cells, glial cells, and endothelial cells. This protein family, which includes VEGF-A through VEGF-D and the placental growth factor (PGF), is upregulated in response to the ischemic and hypoxic conditions triggered by HIF-1. Its pivotal role in DR, DME, and NVG is well documented, particularly its effect in disrupting retinal capillary permeability [149,200,201,202]. Notably, VEGF exacerbates disruptions in retinal capillary integrity by modifying the proteins crucial for maintaining tight junctions, such as zonula occludens. This disruption initiates several downstream pathways such as the mitogen-activated protein kinase (MAP) kinase and phosphoinositide 3-kinase/akt (PI3/AKT) pathways, leading to endothelial cell proliferation and migration [23]. This cascade further stimulates enzymes like MMPs and the urokinase-type plasminogen activator, which facilitate the degradation of basement membranes crucial for new capillary formation [203]. The newly formed capillaries stabilize by recruiting pericytes and smooth muscle cells, a process regulated by PDGF [204].

Concurrently, the NF-κB signaling pathway plays a significant role in inflammation and immune responses within the diabetic eye [205,206]. Activated by various stressors, NF-κB drives the expression of inflammatory mediators such as iNOS and ICAMs, which are critical in the development of retinopathy [206,207]. These mediators facilitate inflammation, leading to apoptosis, leukostasis, and the breakdown of the BRB, which are hallmark features of DR and DME [206,207,208]. Furthermore, NF-κB contributes to delayed corneal healing and exacerbated endothelial damage through oxidative stress and AGEs [82]. In the context of diabetic cataracts, AGEs activate several signaling pathways via the receptor for AGE, promoting oxidative stress and further activating NF-κB, contributing to lens opacification [118,119,125].

Similarly, the JAK-STAT signaling pathway is critical, as it is where STAT proteins such as STAT1, STAT3, and STAT5 regulate cell functions including proliferation, differentiation, apoptosis, and inflammation [22,209]. These proteins are upregulated in diabetes, influencing tight junctions, promoting endothelial cell injury, and exacerbating RPE cell dysfunction, which in turn worsen the integrity of the BRB [20,210,211]. In addition, STAT proteins activate pro-inflammatory microglia and circulating immune cells, leading to capillary occlusion, further worsening BRB disruption [210,212,213,214].

The MAP kinase pathway is integral to numerous physiological processes, such as gene expression, cell proliferation, differentiation, survival, and apoptosis [215]. Specifically, the MAPK/ERK signaling pathway plays a crucial role in various pathological mechanisms, including the regulation of cell cycle progression and chronic inflammation, which is a defining feature of DR [216,217]. ERK activation is particularly important in neuroretinal tissue, where it acts as a neuroprotective agent, supporting the interaction between the neuroretina and the RPE [218,219]. This interaction is essential for maintaining the integrity of the inner BRB. When ERK activity is suppressed, it disrupts the neuroretina–RPE interaction, leading to subretinal fluid accumulation and accelerating the progression of DR [220]. In contrast, research has demonstrated that AGEs and their receptors activate the MAPK/ERK pathway, promoting oxidative stress and triggering the overexpression of pro-inflammatory cytokines, adhesion molecules, and vascular regulators. These processes contribute to retinal lesions and damage vascular endothelial cells [221,222]. Additionally, in diabetic keratopathy, AGEs have been shown to induce apoptosis in human corneal epithelial cells through the generation of ROS and the activation of the JNK and p38 MAPK pathways, exacerbating the cellular damage observed in diabetic eye disease [83].

The PI3K/Akt/mechanistic target of rapamycin (mTOR) pathway is another critical signaling pathway for cell survival and proliferation, particularly in pathological angiogenesis, a hallmark of PDR [223]. In PDR, the typical hypoxic conditions seen trigger the activation of this pathway through growth factors like IGF-1, leading to increased VEGF expression and promoting abnormal neovascularization [224,225]. Moreover, mTOR signaling is vital for the hypoxia-induced proliferation of vascular cells, including smooth muscle and endothelial cells, by increasing their sensitivity to growth factors [226]. In diabetic keratopathy, hyperglycemia inhibits the PI3K/Akt pathway, which is crucial for cellular survival and regeneration. This inhibition further disrupts corneal healing, reducing the capacity of corneal epithelial cells to repair and regenerate effectively [86].

Several targeting drugs have been developed and used to target specific pathways involved in various clinical aspects of DED, providing effective strategies for treating and preventing the progression of diabetes-related ocular disorders (Table 1).

## 10. Conclusions

DED remains a leading cause of visual impairment and blindness worldwide, driven by complex mechanisms including chronic hyperglycemia, inflammation, and oxidative stress. This review highlights the critical role of immune-mediated processes and dysregulated signaling pathways, such as VEGF, NF-κB, JAK-STAT, MAPK, and PI3K/Akt/mTOR, in the pathogenesis of DR, DME, DK, diabetic cataract, and glaucoma. The involvement of both innate and adaptive immune responses, particularly through the actions of the microglia, macrophages, and inflammatory cytokines, underscores the importance of targeting immune pathways for therapeutic intervention. Despite advances in our understanding of these mechanisms, further research is needed to fully elucidate their immunological contributions to DED and identify new therapeutic strategies aimed at mitigating inflammation, restoring tissue integrity, and preserving vision in diabetic patients. By focusing on these immune and signaling pathways, targeted therapies may offer new avenues for the prevention and treatment of DED, ultimately improving patient outcomes and reducing the burden of the disease.

## Figures and Tables

**Table 1:** Overview of pathogenic mechanisms in diabetic eye diseases and corresponding targeting drugs.

Pathogenetic Mechanism	Targeting Drug	Category	References
VEGF-A	Bevacizumab, Ranibizumab, Brolucizumab	Anti-VEGF	[227,228]
VEGF-A, VEGF-B, PLGF	Aflibercept	Anti-VEGF	[229]
VEGF-A, Angiopoietin-2	Faricimab	Anti-VEGF	[229,230]
IL-6, IL-8, MCP-1, ICAM-1, TNF-α, VEGF, ANGPT2, etc.	Triamcinolone Acetonide, Dexamethasone, Fluocinolone Acetonide	Immunomodulatory Therapy	[231,232]
IL-1β	Canakinumab	Immunomodulatory Therapy	[233]
TNF-α	Infliximab	Immunomodulatory Therapy	[35,234]
LFA-1	Lifitegrast	Immunomodulatory Therapy	[235]
VCAM-1 VLA-4	Anti-CD49 antibody	Immunomodulatory Therapy	[236,237]
αVβ3, αVβ5, α5β1, and αMβ2 integrins	Risuteganib	Immunomodulatory Therapy	[238]
IL-6	EBI-031	Immunomodulatory Therapy	Clinical Trial ID: NCT02842541
IL-6 receptor	Tocilizumab	Immunomodulatory Therapy	Clinical Trial ID: NCT02511067
JAK/STAT	JAK inhibitor I, tofacitinib, STAT3 inhibitor	Signaling Pathway Inhibitor	[239]
NF-κB	JSH-23	Signaling Pathway Inhibitor	[206]
MAPK	PHA666859	Signaling Pathway Inhibitor	[240]
PI3K/Akt	Transthyretin (TTR)	Signaling Pathway Inhibitor	[241]
PI3K/Akt	Topical Insulin	Signaling Pathway Inhibitor	[242]
Wnt/β-catenin	Topical Insulin	Signaling Pathway Inhibitor	[243]
IGF-1	Topical Insulin	Signaling Pathway Inhibitor	[244]
AGE	Alpha-lipoic acid	Antioxidant/Neuroprotective Agent	[245]
ROS/NLRP3/caspase-1	N-acetylcysteine	Antioxidant/Neuroprotective Agent	[246]
ROS/NLRP3/caspase-1	Calcitriol	Antioxidant/Neuroprotective Agent	[247,248]
NF-κB	Lutein	Antioxidant/Neuroprotective Agent	[249]
HMGB1, IL-1β, TLR2, TLR4, NLRP3, COX2, SOD2, HO-1, GPX2, GR1, CXCL2, iNOS	Glycyrrhizin	Antioxidant/Neuroprotective Agent	[250]
ROS, RAGE, SOD-1	PEDF	Antioxidant/Neuroprotective Agent	[251]
TNF-α, IL-6, IL-1β, MCP-1, IFN-γ, MMP-2, MMP-9, IL-10, SOD-1	rhFGF-21	Antioxidant/Neuroprotective Agent	[252]
IL-10, TNF-α, IL-6, IL-1β	ARA290	Antioxidant/Neuroprotective Agent	[253]
VEGF	Flt23k	Gene Therapy	[254]
VEGF	small interfering RNA targeting HIF-1alpha and VEGF	Gene Therapy	[255]
VEGF receptor 1	sFLT-1	Gene Therapy	[256]
Neovascularization	PEDF, Endostatin, Calreticulin	Gene Therapy	[257]
Wound healing, immune privilege	Hemopoeitic stem cells	Stem Cell Therapy	[258,259]
Neuroprotection	Autologous Bone Marrow Mesenchymal stem cells	Stem Cell Therapy	[260]

VEGF: Vascular Endothelial Growth Factor; PLGF: Placental Growth Factor; IL-6: Interleukin 6; IL-8: Interleukin 8; MCP-1: Monocyte Chemoattractant Protein-1; ICAM-1: Intercellular Adhesion Molecule 1; TNF-α: Tumor Necrosis Factor Alpha; ANGPT2: Angiopoietin-2; IL-1β: Interleukin 1 Beta; LFA-1: Lymphocyte Function-Associated Antigen 1; VCAM-1: Vascular Cell Adhesion Molecule 1; VLA-4: Very Late Antigen-4; αVβ3: Integrin Alpha-V Beta-3; αVβ5: Integrin Alpha-V Beta-5; α5β1: Integrin Alpha-5 Beta-1; αMβ2: Integrin Alpha-M Beta-2; JAK: Janus Kinase; STAT: Signal Transducer and Activator of Transcription; NF-κB: Nuclear Factor Kappa B; MAPK: Mitogen-Activated Protein Kinase; PI3K: Phosphoinositide 3-Kinase; Akt: Protein Kinase B; Wnt: Wingless-related Integration Site; IGF-1: Insulin-like Growth Factor 1; AGE: Advanced Glycation End-products; ROS: Reactive Oxygen Species; NLRP3: NOD-, LRR- and Pyrin Domain-Containing Protein 3; HMGB1: High Mobility Group Box 1; TLR2: Toll-like Receptor 2; TLR4: Toll-like Receptor 4; COX2: Cyclooxygenase 2; SOD2: Superoxide Dismutase 2; HO-1: Heme Oxygenase 1; GPX2: Glutathione Peroxidase 2; GR1: Glutathione Reductase 1; CXCL2: Chemokine (C-X-C Motif) Ligand 2; iNOS: Inducible Nitric Oxide Synthase; RAGE: Receptor for Advanced Glycation End-products; IFN-γ: Interferon Gamma; MMP-2: Matrix Metalloproteinase-2; MMP-9: Matrix Metalloproteinase-9; IL-10: Interleukin 10; PEDF: Pigment Epithelium-Derived Factor; rhFGF-21: Recombinant Human Fibroblast Growth Factor 21; sFLT-1: Soluble Fms-like Tyrosine Kinase-1.
